# The role of ICOS in the development of CD4 T cell help and the reactivation of memory T cells

**DOI:** 10.1002/eji.200636661

**Published:** 2007-07

**Authors:** Simmi Mahajan, Ana Cervera, Megan MacLeod, Simon Fillatreau, Georgia Perona-Wright, Stephen Meek, Andrew Smith, Andrew MacDonald, David Gray

**Affiliations:** 1 Institute of Immunology and Infection Research, University of Edinburgh,Ashworth Laboratories, King's Buildings, Edinburgh, UK; 2 Unidad Mixta de Investigación Centro Nacional de Investigaciones Cardiovasculares Carlos III – Universitat de Valencia,Valencia, Spain; 3 Institute of Stem Cell Research, University of Edinburgh,King's Buildings, Edinburgh, UK

**Keywords:** B cells, Cell differentiation, Costimulation, Memory, T cells

## Abstract

We have addressed the role of the inducible costimulator (ICOS) in the development of T cell help for B cells and in the generation, survival and reactivation of memory CD4 T cells and B cells. We find that while T cell help for all antibody isotypes (including IgG2c) is impaired in ICOS knockout (ICOS-KO) mice, the IFN-γ response is little affected, indicating a defect in helper function that is unrelated to cytokine production. In addition, the ICOS-negative T cells do not accumulate in B cell follicles. Secondary (memory), but not primary, clonal proliferation of antigen-specific B cells is impaired in ICOS-KO mice, as is the generation of secondary antibody-secreting cells. Analysis of endogenous CD4 memory cells in ICOS-KO mice, using MHC class II tetramers, reveals normal primary clonal expansion, formation of memory clones and long-term (10 wk) survival of memory cells, but defective expansion upon reactivation *in vivo*. The data point to a role of ICOS in supporting secondary, memory and effector T cell responses, possibly by influencing cell survival. The data also highlight differences in ICOS dependency of endogenous T cell proliferation *in vivo* compared to that of adoptively transferred TCR-transgenic T cells.

## Introduction

Inducible costimulator (ICOS) is a costimulatory molecule in the CD28 family, whose expression is induced during the activation of CD4 T cells [1, 2]. Most notably, it is expressed on the T cell subset found in B cell follicles [1]. It is also associated with the production of IL-10 by T cells [1]. ICOS ligand (ICOSL), also known as B7RP-1 [3], B7-H2 [4], or B7h [5–7], is expressed on resting B cells, dendritic cells (DC) and activated monocytes. Genetic deletion of ICOS in mice has clarified some of the issues concerning ICOS function, while confusing others [8–10]. For instance, it became clear that in the absence of ICOS the T-dependent B cell response was impaired such that development of germinal centers was minimal [11] and the secretion of switched isotype antibody responses following immunization was defective. Initially, the defect in the production of cytokines by T cells in the knockout mice was shown to be in the Th2 arm of the response (*e.g.* reduced IL-4), as well as a lack of IL-10, while IFN-γ was relatively unaffected and in some cases increased [8–10]. It was surmised that ICOS costimulated for the differentiation of Th2 cells. Subsequently, the Th2 bias has come into question, with clear evidence that ICOS costimulation is required for both Th1 and Th2 responses [12–18]. The association with the IL-10 response still holds and has strengthened, with the development of IL-10-producing regulatory T cells *in vivo* being found to be ICOS dependent [19, 20].

The confusing picture with respect to the role of ICOS in cytokine responses may be related to the assays used; however, a recent paper by Löhning *et al.* [21] offers an attractive explanation. First, these authors showed clearly that ICOS is expressed by effector T cells of all types, irrespective of the cytokine they secrete. However, importantly, they also show that the level of ICOS expression is correlated with the cytokines produced; thus, T cells expressing high levels of ICOS made IL-10, intermediate ICOS expressors made Th2 cytokines, while those expressing only low amounts made cytokines such as IL-2, IFN-γ or GM-CSF.

Other contradictions also exist, for instance, some authors have found that the migration of T cells into B cell follicles is independent of ICOS [15], while others find that the development of the follicular helper T_FH_ population requires ICOS [22]. More fundamentally, the role of ICOS in primary clonal expansion of CD4 T cells is still contentious. Most initial studies, using *in vitro* stimulation, showed little effect of ICOS blockade or absence. However, more recently, Smith *et al.* [15] have shown that primary antigen-driven clonal expansion of adoptively transferred TCR-transgenic T cells is susceptible to ICOS blockade. In contrast to the primary response, the role of ICOS in the restimulation and clonal expansion of memory B and T cells during secondary responses has received relatively little attention. To address this, we tracked the antigen-specific B and T cell responses in ICOS-deficient mice using fluorescent antigen, phycoerythrin (PE; B cells) or MHC class II tetramers (T cells). We show that while primary expansions are relatively independent of ICOS, the secondary expansion is dependent on ICOS. We also found that although primary expansion of endogenous (polyclonal) CD4 T cells did not require ICOS, the expansion of naive TCR-transgenic T cells after adoptive transfer and immunization was ICOS dependent.

## Results

### T-dependent antibody responses are impaired in these ICOS^–/–^ mice

The ICOS locus was targeted with the dual aim of inserting a reporter (β-gal) and also to knockout the gene in mice homozygous for the deletion. Details of the targeting strategy are provided in the *Materials and methods* section and in Supporting Information Fig. 1. Confirmation that gene targeting had abolished ICOS expression is provided in Supporting Information Fig. 2.

**Figure 1:**
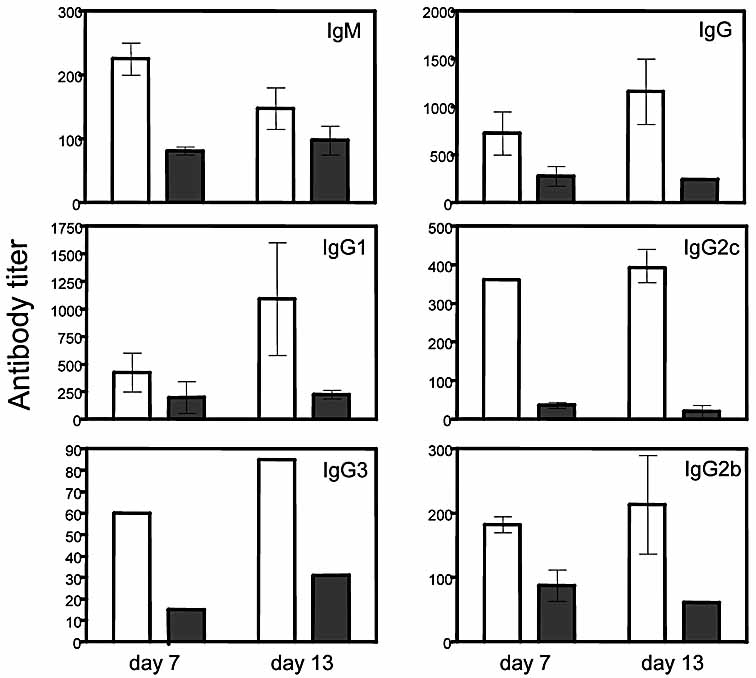
ICOS is critical for production of all IgG subclasses. DNP-specific immunoglobulins in the sera of ICOS^+/+^ (open bars) and ICOS^–/–^ (grey bars) mice at 7 and 13 days after i.p. immunization with alum-precipitated DNP-KLH were measured by ELISA. Antibody titers were calculated by plotting serum concentration against OD and reading the dilution at the half-maximal OD of a positive control. Data is representative of four independent experiments and the error bars indicate the SEM of four mice per group. The data shown here is from mice prior to extensive backcross (generation 5), but is almost identical in mice backcrossed for ten generations to C57BL/6. ICOS^+/+^ mice here are littermate controls.

**Figure 2:**
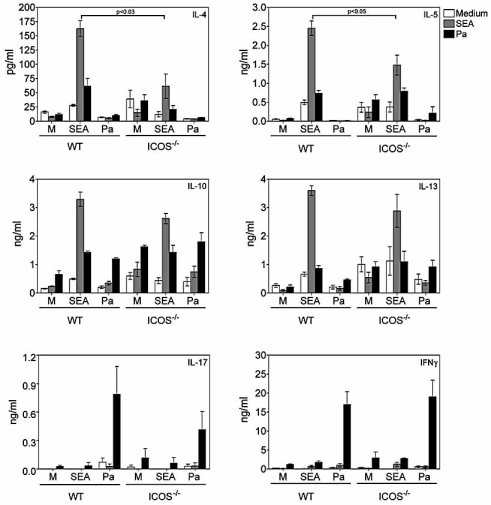
Role of ICOS in effector T cell differentiation. C57BL/6 DC were cultured overnight in medium alone (M), with SEA or Pa and injected into wild-type and ICOS^–/–^ mice i.p. After 7 days, spleen cell suspensions were made and cultured in medium (white bar), with SEA (grey) or with Pa (black) for 3 days before the supernatants were harvested. The levels of cytokine production by splenocytes were measured by ELISA. Data shown is representative of two experiments. Error bars indicate SEM of three to four mice per group. The data shown is from generation 10 C57BL/6 backcross mice.

ICOS^–/–^ and ICOS^+/+^ mice were immunized with haptenated protein, dinitrophenyl-keyhole limpet hemocyanin (DNP-KLH) i.p., and the titer of DNP-specific immunoglobulin isotypes in the sera was measured at days 0, 7 and 13 post immunization by ELISA. As described earlier, a marked defect was observed in the class-switched isotypes in the ICOS^–/–^ mice (Fig. 1). The anti-DNP IgG response was around 2.5-fold lower in ICOS^–/–^ mice at day 7 with a further reduction at day 13 when it was around 5-fold lower. A similar reduction in titers of DNP-specific IgG1 was noticed in the sera of ICOS^–/–^ mice. ICOS^–/–^ mice elicited a markedly reduced IgG2c response both at day 7 (10-fold lower) and day 13 (20-fold lower). Reduction in the titers of IgG3 and IgG2b was notable but less marked than for the other isotypes. Hence, as reported in other ICOS-deficient mice, the interaction between T and B cells *via* ICOS-ICOSL is critical for B cells to produce class-switched antibodies.

Similar to previous reports [11], the ICOS^–/–^ mice had fewer and smaller GC than ICOS^+/+^ mice (Supporting Information Fig. 2).

### Th2 cytokine production is impaired in ICOS^–/–^ mice

We next asked if the secretion of cytokines by CD4 T cells in our ICOS^–/–^ mice correlated with the defects recorded in immunoglobulin isotype production (Fig. 1). The production of cytokines by T cells in response to immunization with either schistosome egg antigen (SEA)-or heat-killed *Proprionebacterium acnes* (Pa)-pulsed DC was measured by ELISA [23, 24]. The data (Fig. 2) are roughly in agreement with published data [8–10], with IFN-γ largely unaffected and Th2 cytokines reduced (IL-4 and IL-5, but not IL-13, reached statistical significance). In the context of the Th1 response, it is interesting to note that, although IgG2c production was dramatically impaired in the antibody response of ICOS knockout mice (Fig. 1), there was no impact of the absence of ICOS on the production of IFN-γ that drives switching to this isotype. Interestingly, the IL-10 response was not reduced as dramatically as expected from previous data. We found that IL-17 was produced only in response to Pa, and although less was made in the restimulation of ICOS^–/–^ T cells, this was not statistically significant.

### ICOS is important for the development of T cell help for B cells

**Figure 3:**
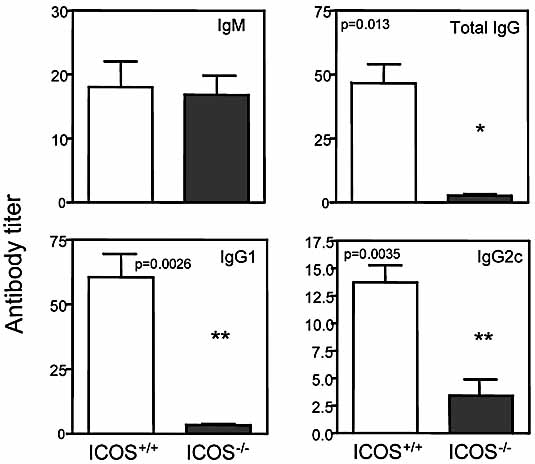
ICOS^–/–^ T cells do not help wild-type B cells to produce antibody. DNP-KLH-primed C57BL/6 B cells were cultured with OVA-primed T cells from ICOS^+/+^ (open bars) and ICOS^–/–^ (grey bars) in the presence of soluble DNP-OVA. The culture supernatant was harvested 7 days later and the amount of DNP-specific antibodies measured by ELISA. Comparative titers were calculated by plotting the concentration against OD and reading the dilution at the half-maximal OD of a positive control. The two groups were compared statistically by unpaired Student's *t*-test. *p <0.05; **p <0.01. Data shown here is from generation 7 C57BL/6 backcross mice and is representative of three independent experiments, each using four mice per group. The error bars indicate the SEM.

The above data suggest that abnormal cytokine secretion is not the basis of the defect in the T-dependent antibody response. To investigate the development of T cell helper function in ICOS^–/–^ mice, we utilized a hapten carrier-based *in vitro* help assay, addressing whether ICOS-deficient T cells can support the production of class-switched antibodies by wild-type B cells. B cells from DNP-KLH-primed C57BL/6 mice were cultured *in vitro* with ovalbumin (OVA)-primed ICOS-deficient or-sufficient purified CD4^+^ T cells in the presence of soluble DNP-OVA. The medium containing the antigen was removed after 2 days and replaced with fresh medium. Culture supernatants were harvested 5 days later and DNP-specific IgM, IgG, IgG1 and IgG2c were measured by ELISA. As shown in Fig. 3, the IgM response was unaffected by the absence of ICOS. However, T cells lacking ICOS were unable to help primed B cells to class switch. The amounts of total IgG and of IgG1 and IgG2c were significantly lower in the supernatants of cultures with ICOS^–/–^ T cells as compared to the ICOS^+/+^ T cells. We conclude that the ICOS-ICOSL interaction between T and B cells is critical for the development of the T cell helper function.

### ICOS^–/–^ CD4 T cells do not migrate into the B cell follicles

**Figure 4:**
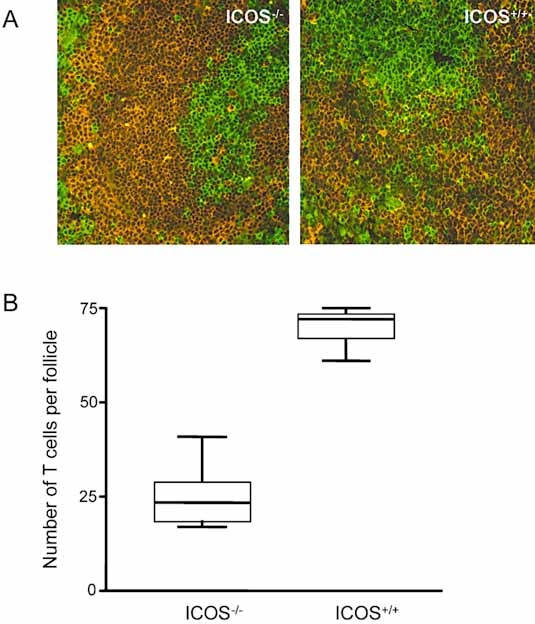
ICOS^–/–^ T cells do not migrate into B cell follicles. (A) Immunohistological stain showing T cells in B cell follicles in ICOS^+/+^ and ICOS^–/–^ mice 8 days after immunization. Note the relative lack of green Thy-1^+^ T cells in the IgM^+^ (red) follicle from the ICOS^–/–^ mouse. Original magnification ×400. (B) The T cell migration was quantified by counting T cells within the IgD^+^ area under the microscope. The data were plotted using a box and whiskers representation. The box extends from the 25^th^ to the 75^th^ percentile, with a horizontal line at the median (50^th^ percentile). Whiskers extend down to the smallest value and up to the largest. The data is a compilation of counts from five mice and is representative of two similar experiments. The data shown is from generation 10 C57BL/6 backcross mice.

T cells provide help to initiate GC, maintain B cell proliferation, cause differentiation within the GC and finally deliver long-term survival signals (*via* CD40) that allow GC B cells to enter the memory pool [25]. These events require the presence of T cells inside or close to B cell follicles. To see if ICOS plays a role in T cell migration, we stained the spleen sections of ICOS^+/+^ and ICOS^–/–^ mice immunized 9 days earlier with DNP-KLH for T and B cells. As can be seen in Fig. 4A, very few CD4 T cells enter the follicles in the ICOS^–/–^ mice. Quantitation revealed that the number of T cells per follicle in ICOS^+/+^ mice was threefold higher than in ICOS^–/–^ mice (Fig. 4B). Quantitation of follicular (IgD^+^) areas showed no difference in follicular size in ICOS^+/+^ and ICOS^–/–^ mice.

### ICOS^–/–^ mice have fewer class-switched antibody secreting cells

**Figure 5:**
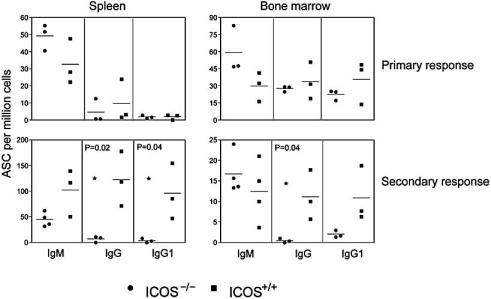
ASC numbers are low in secondary responses in ICOS^–/–^ mice. ELISPOT was performed to enumerate the ASC in spleen and bone marrow of ICOS^+^ (▪) and ICOS^–/–^ (•) mice immunized i.p. with alum-precipitated DNP-KLH 3 wk earlier. Mice were boosted 5 wk after the primary immunization, and spleens and bone marrow were harvested 5 days later to measure the secondary response. Each symbol represents the data from an individual mouse and lines show the mean of each group. Data is representative of two independent experiments using generation 7 C57BL/6 backcross mice. The two groups were compared statistically by unpaired Student's *t*-test; *p <0.05. Representative FACS plots of the secondary response are shown as Supporting Information 3A.

Given that there was impairment to the isotype-switched antibody responses in ICOS^–/–^ mice, we predicted that the number of antibody-secreting cells (ASC) in ICOS-deficient mice would be reduced. To confirm this and to ask if different populations of plasma cells (*i.e.* splenic, short-lived *versus* bone marrow, long-lived) were differentially affected we carried out ELISPOT assays on spleen and bone marrow cell suspensions from immunized mice. ICOS^–/–^ and control ICOS^+/+^ mice were immunized i.p. with alum-precipitated DNP-KLH, and the frequency of DNP-specific ASC in these tissues was measured after 3 wk. Fig. 5 (top panels) shows that the number of ASC producing class-switched antibodies (IgG and IgG1) in the primary response was not significantly different in the ICOS^–/–^ mice in comparison to ICOS^+^ mice, although the trend is toward lower numbers. However, the number of IgM^+^ ASC in the spleen as well as bone marrow was higher in ICOS^–/–^ as compared to control mice. Negligible numbers of class-switched ASC were detected in wells with splenocytes from non-immunized ICOS^–/–^ and ICOS^+/+^ mice (not shown).

To examine if ICOS is required for the secondary ASC response, mice were boosted with DNP-KLH-alum 5 wk after the primary immunization, and spleens and bone marrow were harvested 5 days later. The effect of the absence of ICOS was much more pronounced in the secondary response, as there was a significant reduction in the number of ASC producing IgG and IgG1 in the spleen. A similar trend was noticed in the bone marrow, although the difference was not significant in relation to IgG1. Secondary IgM-producing ASC in the spleen of ICOS^–/–^ mice were notably reduced as well, while this was not apparent in the bone marrow (Fig. 5, bottom panels). The secondary B cell responses were particularly deficient in the absence of ICOS-ICOSL interactions, suggesting a role in the generation or reactivation of antigen-specific memory B cells.

### Antigen-specific B cell expansion in primary and secondary responses in ICOS^–/–^ mice

**Figure 6:**
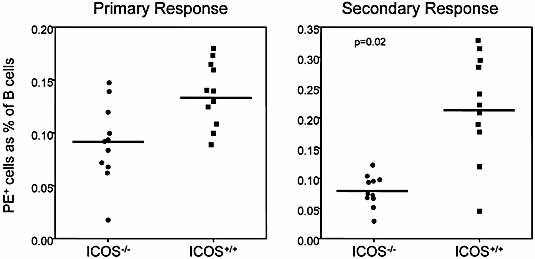
Antigen-specific B cell responses in ICOS^–/–^ mice. Antigen (PE)-driven B cell clonal expansion during primary and secondary response in ICOS^+/+^ and ICOS^–/–^ mice was measured. Splenocytes from mice (generation 10 C57BL/6 backcross) immunized 2 wk earlier with alum-precipitated PE were stained with anti-CD19 antibody and PE. For secondary response, mice were boosted 5 wk later and spleens were harvested 5 days later. PE^+^ cells are plotted as % of CD19^+^ B cells. The data shown is pooled from three independent experiments; it has been analyzed using an unpaired *t*-test, applying Welch's correction for unequal variance for the secondary response.

To investigate the generation of B cell memory in the absence of ICOS, we quantified the expansion of antigen-specific B cells, in primary and secondary responses, by enumerating the PE-binding, CD19^+^ splenic B cells in the mice 2 wk after immunization with alum-precipitated PE. The data shown in Fig. 6 is pooled from three independent experiments, and although there is a trend towards lower antigen-specific expansion in the primary response of ICOS^–/–^ mice, this is not statistically significant.

Mice primed with PE were rechallenged 5 wk later and the secondary expansion of memory PE^+^ B cells was evaluated 5 days after the boost. In this secondary response there was a significant reduction in the percentage of PE^+^ B cells in ICOS^–/–^ mice as compared to ICOS^+/+^ mice (Fig. 6). Thus, secondary/memory B cell clonal expansion requires ICOS and is more dependent than primary clonal expansion.

### CD4 T cell priming *in vivo* using ICOS^–/–^ TCR-transgenic T cells

**Figure 7:**
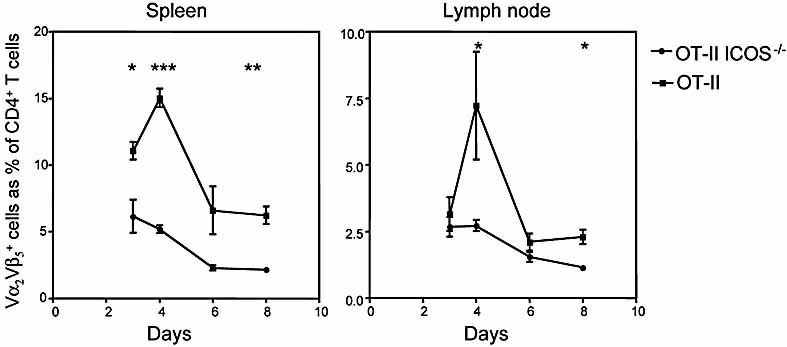
ICOS-dependence of *in vivo* clonal expansion of TCR-transgenic T cells. C57BL/6 mice received OT-II ICOS^+/+^ (▪) or OT-II ICOS^–/–^ (•) lymph node cells 24 h before immunization with OVA-alum i.p., and the expansion of Vα_2_/Vβ_5_^+^ cells was measured at days 3, 4, 6 and 8 in the spleen and inguinal lymph nodes. Vα_2_/Vβ_5_^+^ cells depicted as % of CD4 cells. Both groups were statistically compared using unpaired Student's *t*-test; *p <0.05; **p <0.01; ***p <0.001. The data is representative of three independent experiments, each using four mice per group per time point. The data shown is from generation 10 C57BL/6 backcross mice.

As in previous studies, we found that the primary clonal expansion of T cells, as measured by *in vivo* immunization (with KLH) followed (on days 7 and 13) by *in vitro* antigenic restimulation, did not differ in ICOS^–/–^ or ICOS^+^ mice (data not shown). As this might not reflect the behavior of the T cells *in vivo*, we carried out adoptive transfers of ICOS-deficient and ICOS-sufficient OT-II (OVA peptide-specific, H-2^b^-restricted, TCR-transgenic) lymph node cells into naive C57BL/6 mice, followed by i.p. immunization with alum-precipitated OVA 24 h later. The expansion of CD4^+^ T cells in these mice was followed from day 3 to day 8 post immunization (Fig. 7). In normal mice using this adoptive transfer system, the peak of the clonal expansion is at day 4. We found that T cell priming in the spleen and lymph node was significantly reduced in the absence of ICOS on T cells. The percentage of Vα_2_Vβ_5_-positive CD4^+^ T cells was considerably lower at the peak of the clonal expansion phase when transferred transgenic T cells lacked ICOS (Fig. 7). The kinetics were slightly different also, as the expansion that occurred in the ICOS^–/–^ T cell population (rising from ∼1.6 to 6% in the spleen) peaked at day 3 and not day 4. At the later time point of day 8, corresponding to the end of the contraction phase, the ICOS^+/+^ T cells survived at a higher baseline level compared to ICOS^–/–^ T cells. The behavior of the OT-II T cells in spleen and inguinal lymph node after immunization was broadly similar. This result is in agreement with the study of Smith *et al.* [15] using a similar model.

### CD4 T cell priming *in vivo* in ICOS^–/–^ mice analyzed by MHC class II tetramers

**Figure 8:**
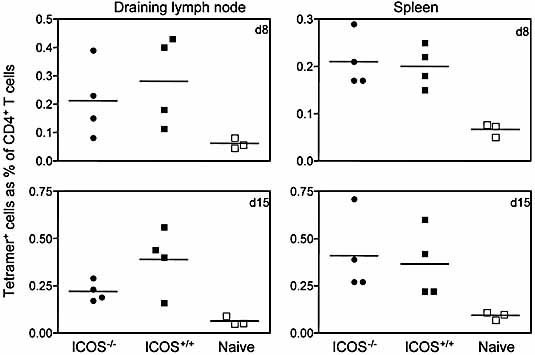
Primary clonal expansion of endogenous T cells is ICOS independent. Antigen (H19-Env peptide)-specific T cells were enumerated using MHC class II tetramers: Tetramer-positive cells expressed as percentage of CD4^+^ T cells in the draining lymph nodes and spleens of ICOS^+/+^ (▪), ICOS^–/–^ (•) and naive (□) mice 8 and 15 days after s.c. immunization with H19-Env-CFA. Ag-specific CD4 T cells detected using IA^b^-H19-Env tetramers. Each point represents an individual mouse and the line shows the mean of the group. Data is representative of two independent experiments using generation 10 C57BL/6 backcross mice.

As the study of memory CD4 T cells using the TCR-transgenic adoptive transfer model is problematic [26, 27], we also followed the endogenous, polyclonal T cell response in normal and ICOS-deficient mice using MHC class II tetramers [28]. The tetramers are H-2A^b^ containing a peptide from the envelope protein (H19-Env) of Moloney murine leukemia virus [29]. As a prelude to the analysis of memory T cells, we investigated priming of the endogenous T cell population. ICOS^–/+^ and ICOS^–/–^ mice were immunized s.c. with H19-Env peptide in CFA and the percentage of tetramer-positive cells was measured at days 8 and 15 in the draining lymph nodes and the spleen. As shown in Fig. 8, the percentage of tetramer-positive CD4 T cells in the spleen as well as the draining lymph nodes at day 8 was equivalent in ICOS^–/–^ and ICOS^+/+^ mice. The same was true also at day 15, although in the lymph nodes of ICOS^–/–^ mice, there were slightly fewer tetramer-positive T cells (not statistically different). We conclude that priming of endogenous CD4 T cells *in vivo* was not impaired in the absence of ICOS signaling.

### Restimulation of CD4 memory T cells is ICOS dependent

**Figure 9:**
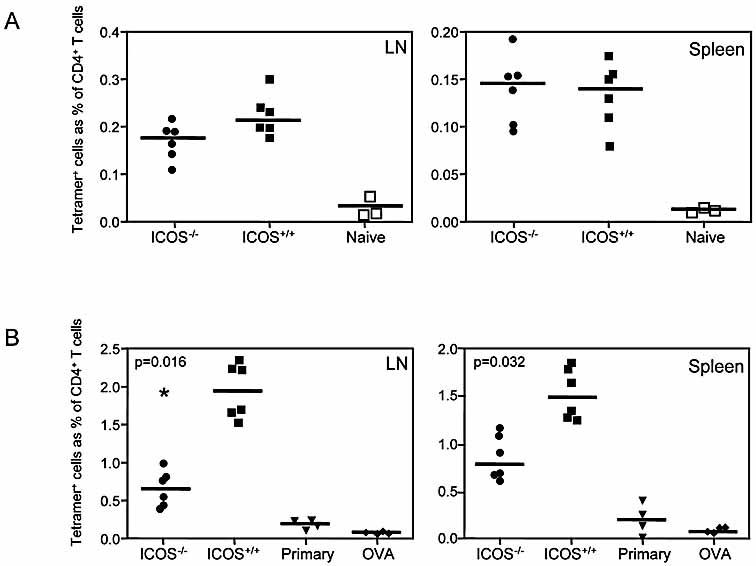
ICOS is needed for reactivation but not survival of memory CD4 T cells. (A) ICOS^+/+^ and ICOS^–/–^ mice were immunized s.c. with H19-Env-CFA and Ag-specific CD4 T cells detected using IA^b^-H19-Env tetramers 10 wk later in the draining lymph nodes and spleen. Each point represents an individual mouse and the line shows the mean of the group. One representative of two independent experiments is shown. (B) ICOS^+/+^ and ICOS^–/–^ mice were immunized s.c. with H19-Env-pulsed, LPS-matured wild-type DC and boosted with peptide-CFA after 10 wk. Mice were killed on day 5 after boost, and draining lymph nodes and spleens taken for tetramer staining. Each point represents an individual mouse and the line shows the median of the group. The data shown is representative of three independent experiments and is from generation 10 C57BL/6 backcross mice. Statistical comparison was done by unpaired Student's *t-*test; *p <0.05. Representative FACS plots of the secondary response are shown as Supporting Information 3B.

Using the MHC class II tetramers, the frequency of antigen-specific CD4 T cells after primary expansion was similar in ICOS^–/–^ and ICOS^+/+^ mice. To test the ICOS dependency of the memory response, we primed mice with H19-Env peptide-pulsed DC and then rested the mice for 10 wk prior to boosting with H19-Env peptide in CFA. Fig. 9A shows that the survival of these antigen-specific memory T cells for 10 wk is unimpaired by the lack of ICOS; thus, prior to immunization, the number of tetramer-positive cells is similar in ICOS^–/–^ and ICOS^+/+^ mice.

Following recall, however, there was a significant impairment of the expansion of the memory population (Fig. 9B). This was particularly evident in the draining lymph nodes and slightly less so in the spleen. So while ICOS is dispensable for the clonal expansion of naive T cells, the proliferation of memory CD4 T cells requires ICOS costimulation.

## Discussion

The expression of ICOS within the first 48 h after T cell activation suggests that it may contribute to the differentiation of T cells during the primary response. Our data suggest that it is not required for the proliferative response, but that it is necessary for the development of the capacity to help B cells make antibody. We have also addressed the role of ICOS costimulation in B and T cell memory responses: The generation of memory B cells and differentiation into antibody-secreting plasma cells in the secondary response is much more affected than the primary response, which is relatively normal. Tracking of the CD4 T cell memory response with MHC class II tetramers shows no impairment of the primary clonal expansion, or long-term survival, but does show impaired secondary expansion. Our data also highlight critical differences in the response requirements of endogenous, polyclonal T cells and adoptively transferred TCR-transgenic T cells.

At first glance, it is tempting to associate the lack of helper function development in ICOS^–/–^ T cells with the defect in cytokine production (especially Th2). However, we are not convinced that differences in cytokine secretion by ICOS^–/–^ T cells underlie this helper defect, for two reasons: First, in the helper assay used here, we are not testing whether the T cells support antibody isotype switching, as this will already have occurred *in vivo* during priming of the B cells in their ICOS-sufficient donor mice. Second, there is a profound lack of both IgG1 and IgG2c (IgG2a equivalent in C57BL/6 mice), the latter being associated with Th1 (IFN-γ) responses, which are quite normal in these mice. What is missing in the *in vitro* assay seems to be the ability to support reactivation and differentiation of the antigen-specific memory B cells to become antibody secretors. This conclusion is supported by the phenotype of the SAP^–/–^ mice [30], which have no particular defect in T cell cytokine production but exhibit reduced IgG1 and IgG2a humoral responses and, interestingly, display impaired expression of ICOS.

What do ICOS-deficient T cells lack as helpers? It could be that they fail to make a particular cytokine (*e.g.* IL-10, IL-6 or, possibly, IL-21) or, possibly, they fail to express surface molecules that provide help/survival signals for B cells. However, ICOS^–/–^ T cells might themselves fail to survive as effector T cells. For instance, the failure of ICOS^–/–^ T cells to populate B cell follicles may not be due simply to a defect in migration of the cells to the follicle, but rather a failure to survive there as a stable population of T_FH_ cells. It will be interesting to see if the helper defect (and follicular colonization) can be rescued by constitutive expression (after retroviral transfection) of survival genes such as bcl-2 or bcl-X_L_. It is worth noting that our current understanding of the sequence of events that lead to follicular localization of T cells does not require ICOS: CD40/CD154 interactions up-regulate OX40L on DC leading to up-regulation of CXCR5 (and down-regulation of CCR7) and hence migration to follicles [31, 32]. A role in survival of activated or effector T cells is consistent with the utilization by ICOS of the phosphoinositol-3-kinase (PI3K) pathway *via* serine/threonine kinases phosphoinositide-dependent kinase 1 (PDK1) and protein kinase B (PKB) to regulate apoptosis [33]. In this regard, there may be convergences in both function and signaling between ICOS and OX40, which mediates survival of effector T cells *via* the PI3K-PKB pathway [34, 35]. This scenario is supported by observations that ICOS blockade *in vivo* is particularly effective when given during the late (effector) phase of Th1/Th17-mediated EAE [14] or Th2-mediated airway inflammation [12]. It has also been observed in less complex *in vivo* models using immunization with nominal antigen that the expansion of already differentiated Th1 or Th2 cells is considerably affected by blocking ICOS [15, 36]. Further evidence comes from studies of Th2 responses in worm infection [37] or in lung inflammation [38, 39], in which ICOS costimulation is needed for the inflammatory response but not for the development of the responding Th2 cells.

The blockade of ICOS in the models of inflammatory disease discussed above [12, 14] was not effective when given during the early priming phase, and there is general agreement that ICOS signaling does not significantly contribute to clonal expansion of naive T cells. However, some experiments have indicated an effect of early ICOS blockade [15]. These studies analyzed the response of adoptively transferred TCR-transgenic cells. Our own analysis of the clonal expansion of small numbers of ICOS-deficient OT-II TCR-transgenic cells transferred into C57BL/6 mice and then immunized with OVA peptide in alum (or CFA) also showed impaired proliferation. In contrast, our analysis using MHC class II tetramers of the primary expansion of endogenous, polyclonal CD4 T cells revealed no defect in ICOS knockout mice. We are left with the conclusion that the requirements for optimal expansion of the transferred population of TCR-transgenic cells are different from those of the endogenous naive T cells. The most likely explanation for this is the difference in initial clone size. Despite transferring only a small number (2 × 10^6^) of transgenic cells, the frequency of antigen-specific naive cells (all with the same TCR idiotope) is at least 100-fold higher than in the endogenous T cell population. It seems likely that stimulatory and/or survival signals (antigen + costimulation) at response initiation are normally limiting and that to support an abnormally large initiating T cell population requires excessive costimulation. We, and many others, have found this TCR-transgenic transfer system problematic with respect to studying memory: The cells do not survive well and cannot be reactivated. Jenkins and colleagues [27] recently showed that the reason for this is the abnormally large clone size of transferred cells and that, when physiologically appropriate numbers of TCR-transgenic CD4 T cells were transferred, the memory clones became stable [27]. The behavior of CD8 memory T cells also seems to be influenced by the original clone size [40]. The results we present here suggest caution in interpreting the data from the TCR-transgenic adoptive transfer models even in the primary response.

To analyze the T cell memory response in ICOS-deficient mice, we utilized MHC class II tetramers and followed the frequency of antigen-specific CD4 T cells over time since primary immunization and then after boosting. There was no difference in the ability of CD4 memory T cells to survive over a 10-wk period in ICOS-deficient or-sufficient mice. However, secondary expansion in ICOS^–/–^ mice was impaired. Likewise, the secondary expansion of memory B cells and secondary ASC in T-dependent responses is much more ICOS dependent than in the primary response. As discussed above, the importance of ICOS in memory responses may simply reflect the increased need for costimulation when antigen-specific precursor frequency is high. However, this interpretation does not fit with the general idea that memory cell restimulation requires less costimulation [41]. Indeed, we recently showed that the proliferation of memory CD4 T cells *in vivo* did not require CD40-CD154 interactions [28]. Thus, ICOS may well be a costimulus that is used preferentially in memory T cell responses. Taken as a whole, our data strongly point to roles for ICOS in supporting effector and memory T cell responses. It will be of future interest to see if these roles utilize a similar mechanism or if they are mechanistically separate.

## Materials and methods

### Mice

ICOS^–/–^, C57BL/6, OT-II [42] and OT-II ICOS^–/–^ mice were bred and maintained under specific pathogen-free conditions in the animal facilities of the School of Biological Sciences at the University of Edinburgh (Edinburgh, UK). Mice were used at 6–12 wk of age, and were sex-and age-matched within experiments as closely as possible. All experiments were covered by a project license granted by the Home Office under the Animal (Scientific Procedures) Act 1986. Locally, this license was approved by the University of Edinburgh Ethical Review Committee.

### Generation of ICOS^–/–^ mice

A knockout allele of the ICOS gene was generated by gene targeting in E14Tg2a ES cells (129/Ola strain) by standard procedures. The targeting vector replaced almost the entire exon 2 and all of the exons 3 and 4 coding sequences with a cassette consisting of a β-galactosidase coding sequence (*lacZ*) preceded by an internal ribosome entry site (IRES) sequence and followed by a neomycin resistance gene expressed from its own promoter (MC1*neo*polyA) (Supporting Information Fig. 1A). Male chimeras generated with ICOS-targeted ES cells were test-crossed with C57BL/6 females and germ-line transmission of the ICOS disruption was confirmed in the agouti coat-colored test-cross offspring by Southern blot analysis of Eco R1-digested DNA obtained by tail biopsy and hybridization with probes flanking each side of the vector integration position (Fig. 1B). Homozygous ICOS^–/–^ mice were generated by inter-crossing male and female test-cross heterozygous ICOS^+/–^ offspring. The ICOS-deficient line has been backcrossed for ten generations to C57BL/6. Some experiments were done on generation 5 and 7 backcross mice (Fig. 1, 3, 5), while the backcrossing was still being carried out, all other experiments were done on generation 10 C57BL/6 backcross mice. The backcross generation used is indicated in the figure legends. Control groups consisted of age/sex-matched C57BL/6 mice.

A cautionary note needs to be added as the region on chromosome 1 that includes the *Icos* gene has been identified as a susceptibility locus for diabetes in NOD mice [43]. Even after ten generations backcrossing to C57BL/6, there remains a significant region around the *Icos* gene that derives from the 129 strain. This region exhibits a polymorphism that influences autoimmune responses [43, 44], although there is no indication in the literature that the 129 strain carries the susceptible locus. Given this and the highly reproducible nature of our results, we are confident of that the effects we observe are the result of the ICOS deficiency.

### Antigens and immunizations

PE (Prozyme), OVA (Sigma-Aldrich), and KLH (Calbiochem) were used as protein antigens. OVA and KLH were coupled to DNP using dinitrofluorobenzene (Sigma-Aldrich). To study B or T cell clonal expansion, the mice were immunized with 200 µg alum-precipitated OVA or 100 µg alum-precipitated PE. OVA was also emulsified in CFA (Sigma-Aldrich) to immunize mice for adoptive transfer experiments. For help assays, mice were injected either with 200 µg alum-precipitated DNP-KLH along with 10^9^ killed *Bordetella pertussis* bacteria (Lee laboratories, Grayson, USA) or 150 µg alum-precipitated OVA with 10^9^ *B. pertussis* bacteria. Endotoxin-free SEA was either prepared in-house [23, 45, 46] or provided by Prof. Mike Doenhoff, University of Bangor. *P. acnes*, a gram-positive bacterium, was obtained from American Type Culture Collection (no. 6919, ATCC, Manassas, VA).

For experiments using MHC class II tetramers, mice were immunized with H19-Env (EPLTSLTPRCNTAWNRLKL) or OVA_323–339_ peptide (ISQAVHAAHAEINEAGR) (both supplied by Advanced Biotechnology Centre, Imperial College, London, UK). The peptides were emulsified in CFA (Sigma-Aldrich) and injected s.c., 100 µg/mouse. In some experiments, peptide-pulsed bone marrow-derived DC were injected s.c.

### Bone marrow-derived DC

For immunization with H19-Env peptide, DC were grown from bone marrow progenitors using GM-CSF, following a protocol based on that of Inaba *et al.* [47]. Bone marrow cells were plated in 24-well tissue culture plates at 3.75 × 10^5^ cells/mL in medium supplemented with 5% GM-CSF containing supernatant from the transfected cell line X63-GM-CSF [48]. Cells were grown for 7 days and washed on days 3 and 6, with the FCS in the medium being replaced with 0.5% mouse serum (Harlan). DC were harvested on day 7, incubated at 37°C together with 0.1 µg/mL LPS (Sigma-Aldrich) at 1 × 10^6^ cells/mL for 12–13 h, and then reharvested. The DC were incubated for 90 min with 50 µg/mL peptide and washed extensively with PBS before injection into mice.

For DC immunization in SEA/Pa experiments, DC were generated by a modified protocol. DC were grown in the presence of recombinant GM-CSF (Peprotech, London, UK) for 11 days, as previously described. Cells were >95% CD11c^+^ MHC class II^+^ DC. To activate the DC, cells were harvested on day 10 of culture and replated at 2 × 10^6^ DC/mL in the presence of either SEA (50 µg/mL) or *P. acnes* [10 µg/mL; both as measured by the Coomassie Plus Protein Assay (Perbio Science UK Ltd., Cramlington, UK)] for their final 18 h of incubation.

### CD4^+^ T cell purification

For *in vitro* help assays, cells from spleens were depleted of B cells, other MHC class II^+^ cells and CD8^+^ T cells to purify CD4^+^ T cells. Following red blood cell lysis, cell preparations were incubated with biotin-conjugated antibodies against MHC class II (M5114, in-house), κ light chain (187.1, in-house), anti-IgM (Southern Biotechnology Associates) and CD8 (53-6.72, in-house) for 15 min on ice. The cells were washed and incubated with streptavidin-conjugated microbeads (Miltenyi Biotech) and then purified on a varioMACS magnet (Miltenyi Biotech) according to the manufacturer's instructions.

### Adoptive transfer of cells

Lymph node cells (popliteal, inguinal, brachial, axillary, superficial cervical, periaortic, iliac and mesenteric) from OT-II mice (ICOS^–/–^ or wild type) were injected i.v. into recipient mice, at 4 × 10^6^–5 × 10^6^ cells/mouse.

### *In vivo* priming by DC

Mice were injected i.p. with 5 × 10^5^ DC that had been cultured for 18 h in the presence of SEA, Pa or medium alone. After 7 days, spleens were removed and 1 × 10^6^ splenocytes plated per well of a 96-well plate in X-vivo 15 serum-free medium (Cambrex, Wokingham, UK) supplemented with 2 mM l-glutamine (Gibco) and 50 µM 2-ME (Sigma). SEA was added to give a final concentration of 25 µg/mL and Pa at 5 µg/mL. Culture supernatants were taken at 72 h to assess their cytokine content by ELISA.

### Flow cytometry

Routinely 10 000 – 20 000 events were acquired on a FACSCalibur flow cytometer running CellQuest software (BD Biosciences), using a live cell gate set by forward and side scatter characteristics. Analysis was performed with FlowJo software (TreeStar). When target populations were small (*e.g.* OT-II adoptive transfers, PE-specific B cells and tetramer^+^ T cells), at least 200 000 events were collected and analyzed. The following antibodies were used: anti-Vα2-PE (BD-Pharmingen), anti-Vβ5-FITC (BD-Pharmingen), anti-CD44-biotin (clone 142.5), anti-CD4-allophycocyanin (APC) (BD-Pharmingen), anti-CD19-FITC (clone ID3) and anti-ICOS-biotin (BD-Pharmingen). In some cases, a secondary reagent, streptavidin-PerCP (BD-Pharmingen), was used.

Mice were immunized i.p. with 100 µg alum-precipitated PE. Spleens were harvested 2 wk following the immunization and cells were stained with 10 µg/mL PE and FITC-labeled anti-CD19 mAb ID3. By the addition of PE, we can detect the PE-binding B cells when analyzed by flow cytometry [49]. PE^+^ cells were compared between groups as percentage of ID3^+^ cells. Background PE staining in un-immunized mice was minimal.

Tetramer staining was done as follows: 1 × 10^6^–2 × 10^6^ erythrocyte-depleted spleen or lymph node cells were plated in 96-well round-bottom plates, washed in IMDM containing 10% FCS, before the addition of PE-labeled MHC class II tetramers diluted in IMDM + 10% FCS. MHC class II tetramers were made and used as described [28]. The cells were incubated at 37°C for 3 h with gentle agitation every 20–30 min before additional antibodies were added: APC-labeled anti-CD4 (BD-Pharmingen), R phycoerythrin (RPE)-Cy5-labeled anti-F4/80 (Serotec) and FITC-labeled anti-CD44 (142.5, in-house), and incubated for 10–15 min at room temperature. Cells were washed three times in FACS buffer (PBS with 2% FCS), and propidium iodide (Pharmingen) was added prior to acquisition. Tetramer-positive cells were identified as CD4 positive, F4/80 negative and propidium iodide negative that bound the H19-Env MHC class II tetramer.

### Immunohistochemistry and quantitative histology

Spleens were frozen in OCT embedding medium (BDH) in cryomoulds (BDH) on dry ice and stored at –80°C. Tissue sections (5 µm) were cut, dried and fixed with acetone. Sections were stained with Texas-red-labeled polyclonal goat anti-mouse IgM (SouthernBiotech) and FITC-conjugated peanut agglutinin (Vector Labs) to visualize the germinal centers. B cell follicles were delineated in spleen tissue sections by staining with anti-IgD antibody (mAb 1126c-biotin). T cells were identified as Thy1^+^ cells (mAb T24-FITC). Slides were viewed on an Olympus BX50 microscope under reflected light fluorescence and images were captured using Openlab imaging software (Improvision). The number of T cells within the IgD^+^ area was counted. For each mouse in a group, 10–15 follicles were counted in this way. The number of follicular T cells was expressed per follicular section. Follicular size was assessed using Openlab software.

### Helper T cell assay

B cells were purified from the spleens of C57BL/6 mice immunized i.p. with alum-precipitated DNP-KLH and killed *B. pertussis*. CD4^+^ T cells were purified from spleens of ICOS^+^ and ICOS^–/–^ mice, immunized with OVA-alum and killed *B. pertussis*. Purified B (10^6^ cells) and T cells (5 × 10^5^ cells) were cultured *in vitro* in the presence of 10 µg/mL soluble DNP-OVA. The medium containing the antigen was removed 2 days later and replaced with fresh medium. The culture supernatant was harvested after 5 days and DNP-specific immunoglobulin isotypes were measured by ELISA.

### Measurement of antigen-specific immunoglobulins by ELISA

Anti-DNP antibodies in the serum of immunized mice or in the culture supernatants of help assays were measured by ELISA. ELISA plates (Dynex Technologies Inc.) were coated with 50 µg/mL DNP-BSA. Doubling dilutions of serum or supernatant were made and incubated for 2 h at room temperature. Following washing in PBS/0.05% Tween-20, plates were incubated with alkaline phosphatase (AP)-conjugated polyclonal goat anti-mouse IgM, IgG, IgG1, IgG2b, IgG2c and IgG3 (all from Southern Biotechnology Associates). Antibody binding was detected by addition of PNPP substrate (SouthernBiotech) to the washed plates and the OD at 405 nm was measured using an ELISA reader (Labsystems). Titers were calculated by plotting serum/supernatant dilution against OD, and taking the half-maximal OD, compared to standard positive serum.

### Measurement of cytokine by ELISA

Maxisorb plates (96-well; NUNC) were coated overnight at 4°C with antibodies against IL-4, IL-5, IL-10, IL-13 and IFN-γ (Sigma-Aldrich). After blocking for 2 h with 10% FCS-PBS, culture supernatant and recombinant cytokine standards (Peprotech or Pharmingen) were added and incubated at 4°C overnight. After washing, cytokine-specific biotin-conjugated antibodies were added (BD Pharmingen) for 1 h at room temperature. Peroxidase-conjugated streptavidin was then added followed by the substrate TMB. Absorbance was measured at 450 nm and the amount of cytokine was measured by interpolation from a standard curve.

### Enumeration of antigen-specific ASC by ELISPOT

Nitrocellulose plates (96-well; Millipore) were coated with DNP-BSA and blocked with 10% FCS-PBS. After washing, spleen and bone marrow cell suspensions were added in triplicate, over the range of 10^4^–10^6^ total cells per well. Following overnight incubation at 37°C, 5% CO_2_, plates were washed thoroughly: three times with PBS, three times with PBS/0.05% Tween-20, then three times again with PBS. The secondary antibody, AP-conjugated goat anti-mouse IgM, IgG, or IgG1 was added in PBS-5% BSA, 4 h at room temperature, then washed. Spots were developed using 5-bromo 4-chlorophosphate (BCIP; Sigma-Aldrich). Spots were scored by eye using a dissection microscope, and the average number of spots per well was used to calculate the frequency of DNP-specific ASC. Spleen and bone marrow from non-immunized control mice consistently gave no spots for IgG, although in one experiment IgM spots were noticed in control mice.

### Statistics

Statistics were calculated using GraphPad Prism version 3.0 (GraphPad software, San Diego, CA). Results were considered significant if *p* <0.05. For some experiments (see figure legends), data from several repeat experiments were pooled and analyzed using an unpaired *t*-test, applying Welch's correction where there were unequal variances.

## Abbreviations:

AP: alkaline phosphatase
ASC: antibody-secreting cell
H19-Env: immunodominant peptide from H19 envelope protein of moloney murine leukemia virus
KLH: keyhole limpet hemocyanin
Pa: heat-killed *Proprionebacterium acnes*
SEA: schistosome egg antigen
